# Proceedings of the Organon Society of Boston

**Published:** 1888-01

**Authors:** S. A. Kimball

**Affiliations:** Secretary


### PROCEEDINGS OF THE ORGANON SOCIETY, BOSTON, MASS.

For some time past there has been a feeling that there were enough homœopathists, in and about Boston, who practiced true Homœopathy, to form a society for mutual advancement in the study of the truths which they endeavor to uphold. This culminated in the following letter of invitation, which was sent to twenty-one physicians in and about Boston:

Boston, December 3d, 1887.

Dear Doctor: We have long thought it would promote the interests of Homœopathy if those who seek to practice it in its purity would draw more closely together for united study, observation, and discussion.

There are twenty-one or two men, including yourself, practicing in or near Boston, who may be said to belong to the class above described, and we invite you to meet them at our office for organization, Thursday evening, December 8th, at 7.45 P. M. It is proposed to call the society the "Organon Society," and to make the study of that work the leading feature of its meetings, and to hold those meetings as often as may be convenient at the homes of members in the city.

You are urgently requested to be present that we may have your advice and co-operation.

Very truly yours,

[Signed], Wm. P. Wesselhœft.

James B. Bell.

There were fifteen out of the twenty-one present December 8th, and those absent sent regrets, with hearty approval of the new departure.

Dr. Wesselhœft in an informal manner explained the object of the gathering, which was to get better acquainted with Homœopathy, and with each other. In no way, he thought, could that be better done than by a careful study of the *Organon*, and as that could better be done in a perfectly informal manner, it did not seem best to organize as a society, but to appoint some one to read each evening, and as points came up for discussion they could be illustrated by cases, and in this manner much practical good would be obtained. In regard to the translation to be used Dr. Wessehœft was of the opinion that that by Dr. Conrad Wesselhœft was by far the best, both in regard to the correctness of the translation and the English used. The only objection was that the foot-notes were in the back of the book instead of at the bottom of the page where they should be. It was thought best to take other translations also for comparison, and in case of doubt to refer to the original in German.

Dr. Bell thought it would be much better to meet in an informal manner, for in that way they would become much better acquainted with each other and not lose so much time in going through the ordinary society routine. He did not think they should be called blind followers of Hahnemann by any means. But having found by experience the value of the truths as laid down in the *Organon*, he thought it would do them all good to go to the fountain head and study once more the truths from which in these days there is so much departure.

After some discussion, in which all present expressed their approval of the ideas advanced, it seemed best, in order to have some regularity to the proceedings, to elect a chairman and secretary, and Dr. W. P. Wesselhœft was elected permanent chairman, and Dr. S. A. Kimball, secretary. It was voted to hold the meetings every other Thursday evening at Dr. Bell's office, for the present, and to have the next meeting December 15th, at 7.45 P. M., and close at 9.30 P. M.

In order to make a good beginning the introduction to the *Organon* was made the subject of the next meeting, and all were requested to be present with their *Organons*.

The first regular meeting of the Organon Society met at Dr. Bell's, Thursday evening, December 15th. Six more invitations had been issued, making the whole number twenty-seven. There were eighteen present in spite of a severe rain-storm.

Dr. Wesselhœft opened the exercises by reading a translation from Bœnninghausen's "Aphorisms" (which is found on another page in the present number). Then the introduction to the *Organon* was commenced. The sixth foot-note was a fertile source of discussion, as to whether coffee should be used in settling the stomach after vomiting; or whether in a paralyzed condition from overeating, sips of strong coffee should be taken in order to strengthen the stomach that it might throw off its contents. It seemed to be the sense of the meeting that while such things were much better than giving emetics, or anything of the sort, the indicated remedy would be one that would be required.

Some interesting remarks were made on the subject of intestinal worms.

Dr. Wesselhœft spoke of two cases in which tapeworms were present—one where the patient, a woman, began to improve from the time the worm was discovered, and from being a very nervous, sickly patient, became healthy and strong. The tapeworm still persisted, and the case going into other hands it was forcibly expelled. From that time her health failed, and in several years' time she died of malignant disease of the stomach. The second case was that of a healthy young lady whose functions were all perfectly normal and who had no symptoms whatever. The only symptom was the presence of the worm, which seemed to have absorbed all the other psoric symptoms in the case. He did not believe they should be expelled even by pumpkin-seed infusion, but the patient should be carefully treated, and when cured the worm would disappear.

Drs. Kennedy and Nichols each spoke of a case in which, under suitable remedies, the worm had entirely disappeared, and no links had been seen for a number of years.

Apropos of worms, Dr. Wheeler related a case of typhoid fever, in which few symptoms were present, and the remedies given had little or no effect. The patient was sick beyond a doubt, for she had a temperature of one hundred and seven degrees for several days. One day he found her sitting up in bed, and in her delirium boring her nose until it bled. Cina was given, and the patient began to improve and finally recovered. On studying Cina it was found to cover beautifully what few symptoms there were in the case, but what pathological homœopath would have thought of Cina in typhoid?

Time being up, the meeting adjourned to meet again December 29th. S. A. KIMBALL, *Secretary.*

